# Supplementary material for: Spatial distribution of suspected and confirmed cholera cases in Mwanza City, Northern Tanzania
Source: PLOS Glob Public Health. 2023 Jan 6;3(1):e0001261. doi: 10.1371/journal.pgph.0001261 (PMC10022096; doi:10.1371/journal.pgph.0001261)
Supplement: Attachment — Submitted filename: Response to Reviewers_October 29 2022.docx [file pgph.0001261.s002.docx]

Journal Requirements:

1. In the online submission form, you indicated that "Aggregated, de-identified data can be obtained by request to the corresponding author.". All PLOS journals now require all data underlying the findings described in their manuscript to be freely available to other researchers, either 1. In a public repository, 2. Within the manuscript itself, or 3. Uploaded as supplementary information.

This policy applies to all data except where public deposition would breach compliance with the protocol approved by your research ethics board. If your data cannot be made publicly available for ethical or legal reasons (e.g., public availability would compromise patient privacy), please explain your reasons by return email and your exemption request will be escalated to the editor for approval. Your exemption request will be handled independently and will not hold up the peer review process, but will need to be resolved should your manuscript be accepted for publication. One of the Editorial team will then be in touch if there are any issues.

**Response:**

Thank you for the clarification, we have omitted the personal identifier to protect our study participants as per the ethical Institutional Ethics Review Board directives. Data used have now been uploaded as suggested.

We further confirm that, the uploaded data we have uploaded into our Supporting Information files, and the information included in our manuscript constitutes our "minimal data set" used to reach the conclusions drawn in the manuscript with related metadata and methods with exceptional to unique personal identification, and any additional data required to replicate the reported study findings in their entirety.

2. Figure 1, 2 & 3: please (a) provide a direct link to the base layer of the map used and ensure this is also included in the figure legend; (b) provide a link to the terms of use / license information for the base layer. We cannot publish proprietary or copyrighted maps (e.g. Google Maps, Map quest) and the terms of use for your map base layer must be compatible with our CC-BY 4.0 license.

**Response:**

We have indicated the map sources. All maps were created by one of the Co-authors (DSKT). We have added this information accordingly.

2. If your map was obtained from a copyrighted source please amend the figure so that the base map used is from an openly available source. Alternatively, please provide explicit written permission from the copyright holder granting you the right to publish the material under our CC-BY 4.0 license.

Please note that the following CC BY licenses are compatible with PLOS license: CC BY 4.0, CC BY 2.0 and CC BY 3.0, meanwhile such licenses as CC BY-ND 3.0 and others are not compatible due to additional restrictions.

Please include your response in the response to reviewers file regarding the above concern or include what you have emailed us previously.

**Response:**

As mentioned in item 1 above, we have indicated the map sources. All maps were created by one of the Co-authors (DSKT). We have added this information accordingly.

3. Please remove the embedded figures from the manuscript file.

For more information about figure files please see our guidelines:

<https://journals.plos.org/globalpublichealth/s/figures> 
<https://journals.plos.org/globalpublichealth/s/figures#loc-file-requirements>

**Response:**

Thank you for the observations, we have revised accordingly. See Fig 1, 2, and 3

4. We have noticed that you have uploaded Supporting Information files, but you have not included a list of legends. Please add a full list of legends for your Supporting Information files after the references list.

**Response:**

Each of the figures and Tables have their sub-title and explanations on their respective pages.

**Additional Editor Comments (if provided):**

In addition to the reviewer comments:

- A stronger take-away message from the study is needed in the abstract (conclusion).

**Response:**

Conclusion have been revised as suggested. See also the conclusion in the main article, page 15, line 329 -334

- In the introduction, there is need to include more details on the knowledge gap that existed (based on available literature on the subject in Tanzania) to justify the need for the study.

- An introductory paragraph is needed in the discussion summarizing the main study findings and giving their broad significance to public health.

**Response:**

We have added this information on page 4, line 76 – 86.

- Avoid brief discussion paragraphs (see page 10, starting on line 221).

**Response:**

The discussion has been expanded as suggested, most of the sub-sections have expanded where possible. See Page 11-15, Line 224 – 320.

- The conclusion needs to be strengthened. Currently, it is majorly a summary of the study findings. What are the implications of the study findings to policy, practice and programming (if at all)?

**Response:**

Conclusion have been revised as suggested. See page 15, line 329 -334

**Reviewer #1**

Authors should take note of the following observations;

1. Introduction: Line 54, rephrasing of sentence needed. "Just one decade later in 2015-16, again? more than....."

**Response:**

Revised to read, **“** Again, in January 2015 to January 2018, 33,421 cases, including 542 deaths were reported, indicating how progressive the trend has been despite various interventions.**” Page 3**

2. Method: more details on the phase 11 study will be appreciated. For example where all the 852 participants mapped from their residences to the various camps? if so, that means you had access to their home addresses. Did the individuals give consent to this information and use of same?

**Response:**

The consent for individual patients was waived as we did not attempt interview or communicate directly regarding data collection. However, in addition to ethics clearance obtained from the BMC/CUHAS Research and Ethics Review Committee (CREC# 129/2016), permission was obtained from the regional administrative Secretary and Mwanza city council. Camp officers in-charges were also asked for their written in informed cost.

It is on this basis personal information related to the study, has not been included in the current Manuscript.

3. **Results:** Kindly give more details on distribution of cholera camps across the two districts surveyed. Reconcile details about mortality in line 144-149 where 6 and 7 were mentioned. If they are different, clearly specify.

**Response:**

Details have been added as suggested, now it reds, “Briefly, in Ilemela District there were four camps (*Viz.,* Igoma (n=298, 34.98 %), Pasiansi (n=165, 19.37%), Sangabuye (n=148, 17.37%) and Bezi (n=14, 1.64%), whereas in Nyamagana District there was only one camp (*Viz.,* Karume (n=227, 26.64%). Few people died (n=7, 0.80%) and only two patients were referred to a hospital.”

Reconciled as suggested; now it reads “Again, in January 2015 to January 2018, 33,421 cases, including 542 deaths were reported, indicating how progressive the trend has been despite various interventions”.

4. **Discussion**: Line 192....corroborate studies? or with? line 208, "10% attended more than 24 hours from onset of symptoms" I think you need to check that again. same as line 221-223, it may need rephrasing

**Response:**

This has been rephrased accordingly, the term “consistence” has been used instead of corroborate, see page, 10. The disease distribution based on age warrants significant economic concerns, as higher confirmed cases were among the working age population (19-60 years); which is consistence with other studies from other African countries such as Nigeria [30] and Uganda [31].

**Line 229**, statement regarding cholera in Northern part near Lake Victoria should be added to section where water source closeness in relation to cholera incidence was mentioned (196-200) for better organization. same as line 233, well and tap water user has been mentioned before (203), merge both line 255-256...rephrase sentence.

**Response:**

We have move and rephrased the mentioned sentences/lines to harmonize the content as suggested. See Page 10 – 11.

5. **Reference**: check 1,5 and 7. when referencing website, date accessed is usually included 30, add journal or website

**Response:**

All references have been revised as suggested. See page17 - 28.

6. **Table**: kindly recheck Table 4, what p-value is considered significant from the analysis you did? this was not mentioned in the methods. from the confidence intervals in the results, p values of 0.10 are not significant with the single asterisk. If you think otherwise, kindly provide more information to the conclusion in method-data analysis section. & Supporting Information: I successfully downloaded the file and opened it, but could not see data on the file. kindly check again.

**Response:**

Thank you for this observation, we have revised Table 4 accordingly. Values with p-value <.05 are considered significant (See page 24 of the Revised Manuscript)

**Reviewer #2:**

1. This paper has the potential to be published because it provides epidemiological insights on the cholera outbreak in a specific region in Tanzania. However, in the introduction I miss why the mapping of the disease was important. Also, I am missing strong implications and recommendations that the local authorities can implement to resolve or prevent any future cholera outbreaks.

**Response:**

This has been added accordingly, See page 296 - 312

2. Please rewrite the final part of the introduction sentence. I am missing a problem statement that will naturally lead to the aim and the objectives of this research paper and why this research was necessary to be conducted.

**Response:**

This has been added, Page 3, line 63 – 65, also Line 76 – 86; and Line 93 – 96.

3. - **Methods**: I don’t understand why the wards are mentioned. Please elaborate.

**Response:**

Wards are low level administrative units used for tracing and possible interventions.

- L84- 92 should be in the introduction section.

**Response:**

Moved to Introduction, see page 4 – 5, line 85 – 95.

- L95 rename to eligibility criteria

**Response:**

We have revised the sun=b-heading to include “Eligibility” See Page 6, Line 115.

- Separate subheading for data analysis

This has been separated as suggested

**Response:**

This has been separated accordingly, see the sub-heading on PAGE 7, Line 138, & 151

- Please create subheadings to make reading the results easier.

**Response:**

This has been separated as suggested, See line 164, 185, and 199

- Please create a separate subheading for limitations and strengths in the discussion section

**Response:**

This has been separated as suggested
