# Supplementary material for: Spatial distribution of suspected and confirmed cholera cases in Mwanza City, Northern Tanzania
Source: PLOS Glob Public Health. 2023 Jan 6;3(1):e0001261. doi: 10.1371/journal.pgph.0001261 (PMC10022096; doi:10.1371/journal.pgph.0001261)
Supplement: Attachment — Submitted filename: Response to Reviewers.docx [file pgph.0001261.s003.docx]

**November 28, 2022**

**PLOS Global Public Health**

To the Editor,

Thank you for the opportunity to revise our manuscript and incorporate the valuable suggestions that the reviewers have made.

We have now addressed the minor changes regarding Table 4 as indicated in our responses below.

Sincerely,

**Dr. Elias Nyanza**

Department of Environmental and Occupational Health, Catholic University of Health and Allied Sciences

Journal Requirements:

1. May the authors address the minor comment on Table 4.

**Reviewer #1:** Authors have effected most observations seen in the original manuscript. However, Table 4 still need review. The notes under the table in the manuscript with track changes and that without track changes are different. The notes with manuscript without tract changes have two asterisks with same interpretation. kindly recheck.

**Response:**

We have effected changes on Table 4. Single asterisk is meant for p ≤ 0.05, whereas double/two asterisk is meant for p ≤ 0.01.

**See Page 33 line 677** in the “Revised manuscript with Track Changes”, or **page 24, line 486** in the clean manuscript.
